# Vorinostat Induces Reactive Oxygen Species and DNA Damage in Acute Myeloid Leukemia Cells

**DOI:** 10.1371/journal.pone.0020987

**Published:** 2011-06-10

**Authors:** Luca A. Petruccelli, Daphné Dupéré-Richer, Filippa Pettersson, Hélène Retrouvey, Sophia Skoulikas, Wilson H. Miller

**Affiliations:** Lady Davis Institute for Medical Research, Segal Cancer Center, Jewish General Hospital, McGill University, Montreal, Canada; Cleveland Clinic, United States of America

## Abstract

Histone deacetylase inhibitors (HDACi) are promising anti-cancer agents, however, their mechanisms of action remain unclear. In acute myeloid leukemia (AML) cells, HDACi have been reported to arrest growth and induce apoptosis. In this study, we elucidate details of the DNA damage induced by the HDACi vorinostat in AML cells. At clinically relevant concentrations, vorinostat induces double-strand breaks and oxidative DNA damage in AML cell lines. Additionally, AML patient blasts treated with vorinostat display increased DNA damage, followed by an increase in caspase-3/7 activity and a reduction in cell viability. Vorinostat-induced DNA damage is followed by a G2-M arrest and eventually apoptosis. We found that pre-treatment with the antioxidant N-acetyl cysteine (NAC) reduces vorinostat-induced DNA double strand breaks, G2-M arrest and apoptosis. These data implicate DNA damage as an important mechanism in vorinostat-induced growth arrest and apoptosis in both AML cell lines and patient-derived blasts. This supports the continued study and development of vorinostat in AMLs that may be sensitive to DNA-damaging agents and as a combination therapy with ionizing radiation and/or other DNA damaging agents.

## Introduction

Histone Deacetylases (HDACs) catalyze the removal of acetyl groups from ε-lysine residues of histones resulting in chromatin condensation and gene silencing. HDAC overexpression and aberrant recruitment to the promoters of genes implicated in differentiation and tumor suppression has been reported in a number of malignancies [1], [2]. HDACs can be divided into four classes: class I (HDAC1, 2, 3, and 8), class IIa (HDAC4, 5, 7, and 9), class IIb (HDAC6 and 10), class III (SIRT1, 2, 3, 4, 5, 6, and 7) and class IV (HDAC11) [3]. Class I, II and IV are zinc-dependent deacetylases, while Class III is NAD^+^-dependent [3], [4]. HDACs can also remove acetyl residues from non-histone proteins, thereby altering their function, providing another mechanism by which HDACs may participate in the malignant phenotype. Targeting HDAC activity using pharmacological small molecule HDAC inhibitors (HDACi) has become an exciting therapeutic strategy. Early studies correlated the anti-tumor effects of HDAC inhibition with restoration of expression of genes involved in differentiation and tumor suppression [5], [6]. However, subsequent microarray studies found that HDACi directly or indirectly activate only 7–10% of all genes, with roughly the same number repressed [7]. This suggested that the anti-tumor activities of HDACi were not limited to their effects on transcription. Indeed, a recent study found 1750 proteins to be acetylated in response to HDACi [8], further suggesting that HDACi can influence diverse cellular pathways.

While HDACi were initially associated with differentiation therapy [9], [10], recent work with HDACi has expanded into understanding mechanisms of HDACi cytotoxicity. All HDACi have been reported to arrest growth and to activate the extrinsic and/or the intrinsic apoptotic pathways [3]. Additionally, HDACi have been shown to inhibit tumor growth in animal models [11], [12]. One mechanism by which HDACi are thought to cause cancer cell apoptosis is through the induction of DNA damage and genomic instability [13]. This has been shown to occur through the generation of reactive oxygen species (ROS), deregulation of repair proteins by acetylation and repression of DNA damage repair protein expression [13], [14], [15]. It is worth mentioning that normal cells have been reported to be significantly less sensitive than tumor cells to all these effects [16].

Vorinostat (suberoylanilide hydroxamic acid, Zolinza™) is a broad spectrum HDACi that inhibits zinc-dependent HDACs (Class I, II and IV). Vorinostat received FDA approval for use in Cutaneous T-cell lymphoma (CTCL) and is in clinical trials, both as a single agent and in combination with other agents in a number of other malignancies [17]. It has also been shown to induce ROS and DNA damage in a number of tumor types [13], [18]. Nonetheless, the characterization of vorinostat in acute myeloid leukemia (AML) cells remains incomplete.

Vorinostat has previously been shown to induce reactive oxygen species, growth arrest and apoptosis in leukemia cells and leukemia mouse models [19], [20], [21], [22]. However, the ability of vorinostat to induce DNA damage in leukemia cells is yet to be reported. In this study, we investigated the mechanisms by which vorinostat-induced DNA damage affects cell growth and apoptosis. We show for the first time that at low, clinically achievable doses, vorinostat induces a variety of DNA damage in leukemia cell lines and AML patient-derived blasts cultured *ex vivo*. These data demonstrate DNA damage to be a contributor to vorinostat-mediated growth arrest and apoptosis mechanisms.

## Materials and Methods

### Ethics Statement

Patient samples obtained from the Quebec Leukemia Cell Bank were collected in accordance with the ethical requirements and regulations of the Quebec Leukemia Cell Bank Ethics Committee. Ethical approval for this study was obtained from the Jewish General Hospital and the McGill Institutional Review Board. All patient samples obtained, collected and used were done so in accordance to the ethical requirements and regulations of the Jewish General Hospital and the McGill Institutional Review Board. Informed written consent was obtained from all participants involved.

### Cell lines, patient cells and reagents

Vorinostat was obtained courtesy of Merck (Whitehouse Station, NJ, USA) and dissolved in DMSO. Z-VAD-FMK was purchased from Promega. N-acetyl cysteine (NAC) was purchased from Sigma and dissolved in DMEM. NB4 and U937 cells were obtained from ATCC and cultured in RPMI supplemented with 10% fetal bovine serum (FBS) at 37°C with 5% CO_2_. Peripheral blood mononuclear cells (PBMCs) from healthy volunteers and blasts from patient FG04290 (AML type M3) and 281010 (AML inv(3)) were isolated from blood samples obtained with patient informed consent. Briefly, blood was centrifuged and the serum discarded. Cells were then resuspended in HBSS, layered over Ficoll and centrifuged. Blasts that remained suspended in the Ficoll were collected for cell culture and treatment. Cryopreserved blasts from patients 03-H070 and 07-H020 (type M3) were obtained from the Quebec Leukemia Cell Bank (Maisonneuve-Rosemont Hospital).

### COMET assay

COMET assays was performed under alkaline or neutral conditions as previously described [23]. Briefly, cells were imbedded in low melting point agarose and layered onto glass slides (Trevigen). Slides were incubated in lysis buffer overnight at 4°C. Slides were then washed and incubated in alkaline buffer (pH>13) for 30 minutes. The COMET slides were then subjected to electrophoresis at 20 V and 240 mA for 30 minutes. Afterwards, the slides were washed in distilled water and fixed using 70% ethanol. Finally, cells were stained with ethidium bromide (1 mg×mL^−1^). Images were randomly collected for analysis of at least 100 nuclei in duplicate experiments using a fluorescent microscope (Leica). Comets were scored using CometScore (TriTek) for tail moment (TM) and tail length (TL). TM and TL are presented in arbitrary units (a.u).

### Western analysis

Whole cell extracts were prepared from pelleted cells lysed in RIPA buffer (150 mM sodium chloride, 1.0% Triton X-100, 0.5% sodium deoxycholate, 0.1% SDS, 50 mM Tris pH 8.0). Western blot was performed on extracts to detect protein levels of γH2AXser139, NBS1 (Cell Signaling), Ku70, cyclin-E and RAD51 (Santa Cruz Biotechnology). β-Actin was purchased from Sigma and confirmed equal protein loading.

### BrdU-PI staining

BrdU was added to cells in culture for 15 minutes, collected, washed twice in warm PBS, and fixed in ice cold 70% ethanol. Fixed cells were then washed twice in PBS and once in PBS with 0.1% BSA. Finally, cells were incubated with BrdU-FITC conjugated antibody, washed and counterstained with propidium iodide. Samples were analyzed using a FACSCalibur flow cytometry (BD Bioscience) and FCS Express v3.0 (De Novo Software). Ten thousand events were collected per sample.

### Quantification of γH2AXser139 and P-Ser10-H3 by flow cytometry

Cells were treated and fixed in 1% paraformaldehyde. Afterwards, cells were permeabilized by 1% Triton dissolved in PBS. Permeabilized cells were incubated with a FITC-conjugated antibody directed against γH2AXser139 or P-Ser10-H3 at room temperature. Cells were concomitantly stained with propidium iodide to assess DNA content. Fluorescence was measured by flow cytometer and analyzed using FCS Express v3.0. Ten thousand events were recorded per sample.

### Propidium iodide staining

Cells were seeded at 2×10^5^cells×mL^−1^ and treated as described. Subsequently, cells were collected and washed in supplemented PBS (5% FBS/0.01 M NaN_3_) at 4°C, pelleted, and resuspended in a hypotonic fluorochrome solution (50 µg·ml^−1^propidium iodide, 0.1% sodium citrate, 0.1% Triton X-100). Fluorescence was detected by flow cytometry and analysis was performed using FCS Express v3.0. Ten thousand events were recorded per sample and allowed for investigation of cell cycle and apoptosis. Cells undergoing DNA fragmentation and apoptosis were defined as events with fluorescence weaker than the Go-G1 cell cycle peak (Sub-Go).

### ROS Detection

Cells were treated with vorinostat for 6 h and 18 h. Cells were then incubated with 25 mg/ml 2′-7′-dichlorofluorescin diacetate (DCF-DA) for 30 minutes at 37°C for the detection of intracellular peroxides (mainly H_2_O_2_). Cells were collected and washed in PBS. Fluorescence was measured by flow cytometry and analyzed using FCS Express v3.0.

### Cell viability and caspase-3/7 assay

Cell viability was assayed using CellTiter-Glo® Luminescent Cell Viability Assay (Promega). This assay aims to quantify the amount of ATP present as this correlates with the number of metabolically viable cells in culture. Briefly, cells treated with vorinostat were incubated in a cell lysis solution containing a luciferin derivative, Ultra-Glo™ Recombinant Luciferase and Mg^2+^ for 10 minutes at room temperature. The solution lyses the cells and converts the luciferin derivative into a luminescent signal proportional to the amount of ATP present. Caspase-3/7 activity was assayed using Caspase-Glo 3/7® Assay (Promega). Briefly, cells treated with vorinostat were incubated in a cell lysis solution containing a luciferase substrate derivative, Ultra-Glo™ Recombinant Luciferase and Mg^2+^ for 1 hour at room temperature. The luciferase substrate derivative is specifically cleaved by active caspase-3/7 resulting in the conversion of the substrate into a luminescent signal (RLU). Luminescence was measured using a FLUOstar OPTIMA microplate reader (BMGLabtech). The luminescent signal was then normalized to cell number and is reported as RLU/cell.

### Glutathione Quantification

Glutathione (GSH) was quantified using GSH-Glo Glutathione Assay (Promega). Briefly, cells were counted and washed with PBS. For each condition, 10 000 cells were plated. Cells were then incubated in a solution containing a luciferin derivative and Glutathione S-Transferase for 30 min at room temperature. In the presence of GSH, the luciferin derivative is converted to luciferin. The cells were then incubated with luciferase enzyme, which initiates a luminescence signal directly proportional to the luciferin previously generated. Luminescence was measured using a FLUOstar OPTIMA microplate reader. Serial dilution of a GSH standard solution was included in order to generate a standard curve, facilitating the conversion of RLU to GSH concentration. Cells treated with buthionine sulphoximine (BSO) were also included as a control.

### Statistics

Significance was determined by ANOVA followed by Newman-Keuls post-tests using Prism version 4.0 (GraphPad).

## Results

### Vorinostat induces a variety of DNA damage in AML cells

**Figure 1:**
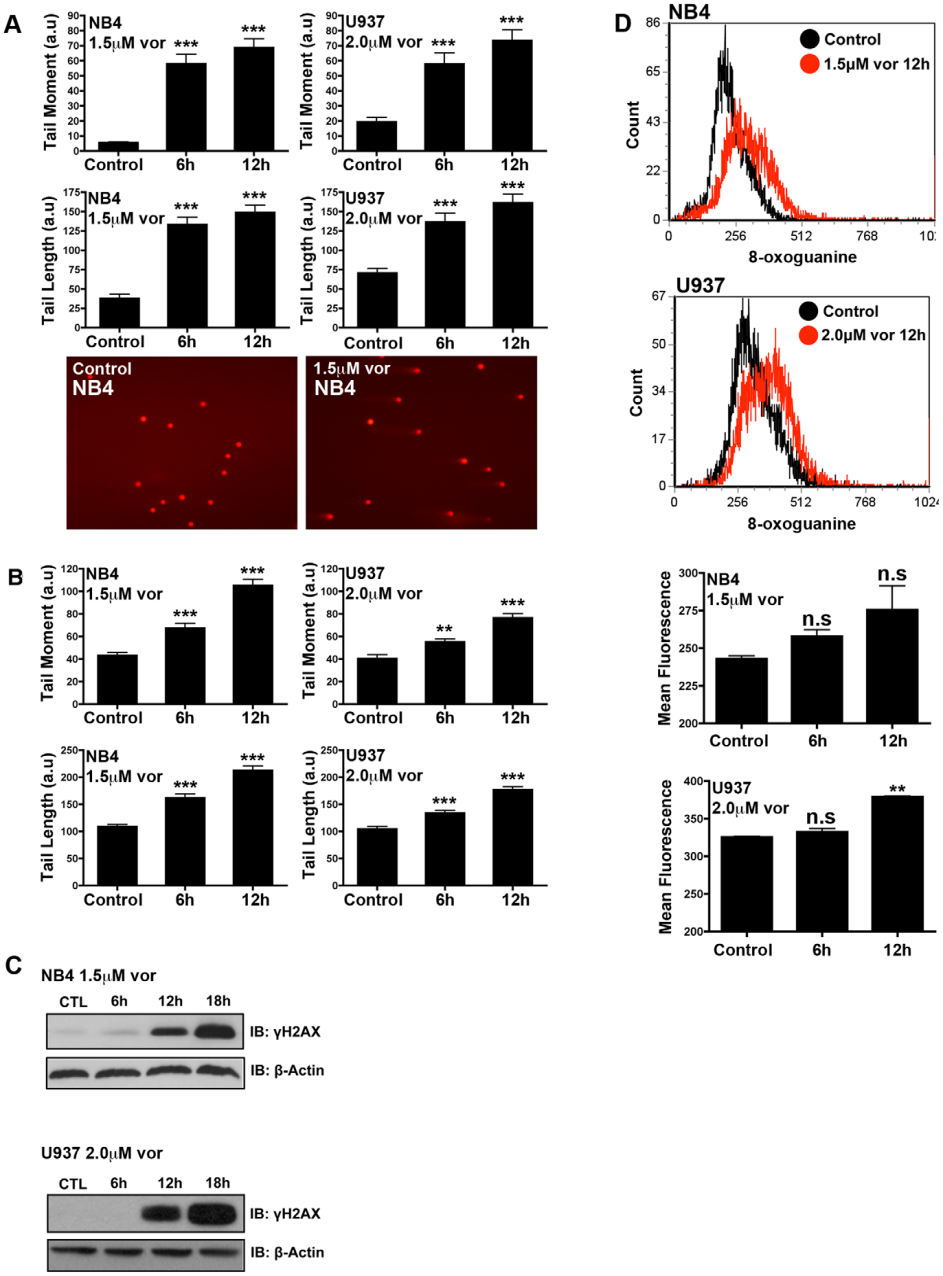
Vorinostat induces different types of DNA damage in NB4 and U937 cells. NB4 and U937 cells were treated with 1.5 µM and 2.0 µM vorinostat, respectively, for the indicated time points. **A** Alkaline COMET assay to measure DNA double strand breaks, single-strand breaks and alkalile-labile sites. At least 100 nuclei were randomly selected and quantified for DNA damage, represented as an increase in Tail Moment (a.u) and Tail Length (a.u). Representative images of COMET tails obtained are presented in the lower panel. **B** Neutral COMET assay to measure DNA double strand breaks. DNA double strand breaks are represented as Tail Moment (a.u) and Tail Length (a.u). **C** Time-dependent induction of γH2AX in response to vorinostat as measured by western blot. β-Actin was used as a loading control. **D** Induction of the oxidative DNA lesion 8-oxoguanine in response to vorinostat was quantified by flow cytometry using a FITC-conjugated antibody. Asterisks show significant difference and a non-significant difference is indicated as “n.s”. (** <0.01 *** <0.001).

Previously, vorinostat has been shown to induce DNA double strand breaks (DSBs) [24]. To investigate the possibility of other types of DNA damage, we performed a comprehensive analysis of vorinostat-induced DNA damage. Two AML cell lines, NB4 and U937, were treated with equally cytotoxic and clinically achievable concentrations of vorinostat before being subjected to single-cell gel electrophoresis (COMET assay). The COMET assay was performed under alkaline conditions, as this permits the detection of a broad spectrum of DNA lesions including DSBs, DNA single-strand breaks and alkalile-labile sites. Treatment with vorinostat caused a significant increase in COMET tail moment and tail length (Figure 1A). To specifically assess vorinostat-induced DSBs, the COMET assay was also performed under neutral conditions, which makes the assay more specific to DSBs but still allows for the detection of cross-links. Indeed, a significant increase in tail moment and tail length was observed and increased in a time-dependent manner (Figure 1B). To verify induction of DSBs, phosphorylation of H2AX was measured by western blot. The phosphorylated form of H2AX (γH2AX) localizes to DSBs within minutes of their formation and is therefore a sensitive marker for this lesion [25]. Vorinostat induced γH2AX after 12 h of treatment in both NB4 and U937 cells (Figure 1C). It should be noted that an increase in γH2AX could be observed after 6 h of vorinostat when blots were over-exposed (data not shown). Also, a number of studies have shown that vorinostat induces ROS. Therefore, we assayed for oxidative DNA damage by staining cells with a fluorescent antibody that recognizes 8-oxoguanine (8-oxo-G) and quantified its induction by flow cytometry. This DNA adduct is a sensitive marker of oxidative stress. We detected an early and time-dependent increase of 8-oxo-G in both NB4 and U937 cells (Figure 1D). Collectively, these data demonstrate that vorinostat is able to induce a variety of DNA damage including DSBs and oxidative DNA lesions.

### Vorinostat increases DNA replication and induces a G2-M arrest followed by apoptosis

**Figure 2:**
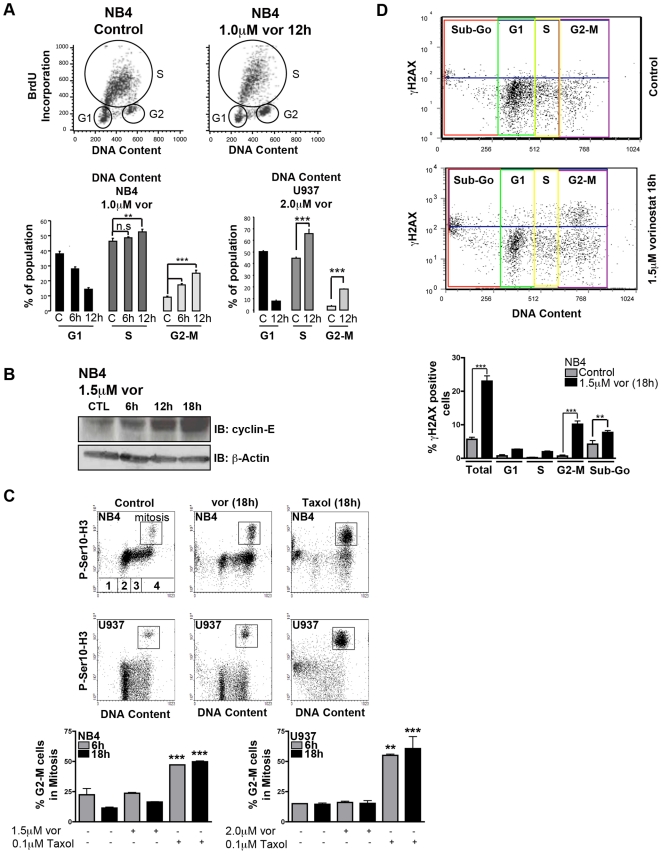
Vorinostat causes cells to arrest in the G2-M phase of cell cycle and arrested cells have accrued DNA double strand breaks. **A** Cells were double stained with BrdU and PI, and analyzed by flow cytometry. Graphs show quantification of cells in each phase of the cell cycle: cells arrested in G1 phase (BrdU negative; 2N DNA content), in S phase (BrdU positive) and in G2-M phase (BrdU negative; 4N DNA content). **B** Western blot analysis of cyclin-E expression in NB4 cells treated with vehicle or 1.5 µM vorinostat. β-Actin was used as a loading control. **C** Cells were double stained with an antibody against P-Ser10-H3 and PI, and analyzed by flow cytometry. For DNA content, 1 = Sub-Go, 2 = G1, 3 = S and 4 = G2-M. Dot plots are representative of data collected and show an increase in mitotic cells positive for P-Ser10-H3, and an accumulation of cells in G2-M. Graphs show that the ratio of P-Ser10-H3 positive cells to cells with 4N DNA content (G2-M) does not change in response to vorinostat. **D** Cells were double stained for γH2AX and PI. Dot plots are representative of data collected and show an increase in γH2AX positive cells that is most significantly increased in G2-M. The graphs demonstrate an increase in the percentage of γH2AX positive cells amongst the cells arrested in the G2-M cell cycle phase. Asterisks indicate a significant difference and a non-significant difference is indicated as “n.s” (** <0.01 *** <0.001).

DNA damage is often accompanied by arrest in cell cycle, and HDACi have been shown to cause a G2-M and/or G1 arrest depending on the malignancy/model and HDACi concentration used. To determine if vorinostat-induced DNA damage resulted in a cell cycle arrest, we performed a double-stain with BrdU (incorporates into replicating cells) and PI (provides a measure of DNA content). The BrdU-PI stain allows for gating and accurate calculation of the percentage of cells in each phase of the cell cycle and discriminates late S-phase from G2 cells and early S-phase cells from G1, as opposed to only staining with PI. We observed a substantial increase in BrdU negative cells with 4N DNA content (G2-M phase) after 6 h–12 h of vorinostat with a decrease in BrdU negative cells with 2N DNA content (G1 phase) (Figure 2A). This demonstrates that vorinostat induces a G2-M phase arrest consistent with the appearance of DNA damage.

We detected a slight increase in replicating cells (BrdU positive cells), which is supported by an increase in cyclin-E as detected by western blot (Figure 2B). To further characterize the G2-M arrest, a double-stain with histone H3 phosphorylated at serine 10 (P-Ser10-H3), a marker of mitosis, and PI was performed and quantified by flow cytometry. Vorinostat was able to induce P-Ser10-H3 and, as previously shown, caused an accumulation of cells with a G2-M DNA content (Figure 2C). However, there was no change in the ratio of P-Ser10-H3 positive cells to cells arresting in G2-M. This suggests that the cell cycle arrest is not specific to the G2-phase or mitosis. Taxol was used as a positive control, as it stabilizes microtubules, causing cells to arrest in mitosis resulting in a significant increase in the percentage of P-Ser10-H3 positive cells within the G2-M population [26]. Next, to determine if the cells arresting in G2-M have damaged DNA, a double-stain with γH2AX and PI was performed and quantified by flow cytometry. The γH2AX-PI stain demonstrated that vorinostat induces a significant increase of γH2AX positive cells arresting in G2-M versus vehicle treated control (Figure 2D). This suggests that cells with DSBs eventually arrest and accumulate in the G2-M phase. Importantly, the γH2AX positive cells were not predominantly found in the Sub-Go population. This indicates that γH2AX is not induced solely as a consequence of apoptosis. These data suggest that vorinostat causes cells to exit the G1 phase of the cell cycle and that cycling cells from G1 eventually arrest in the G2-M phase, likely due to an accumulation of DNA damage.

**Figure 3:**
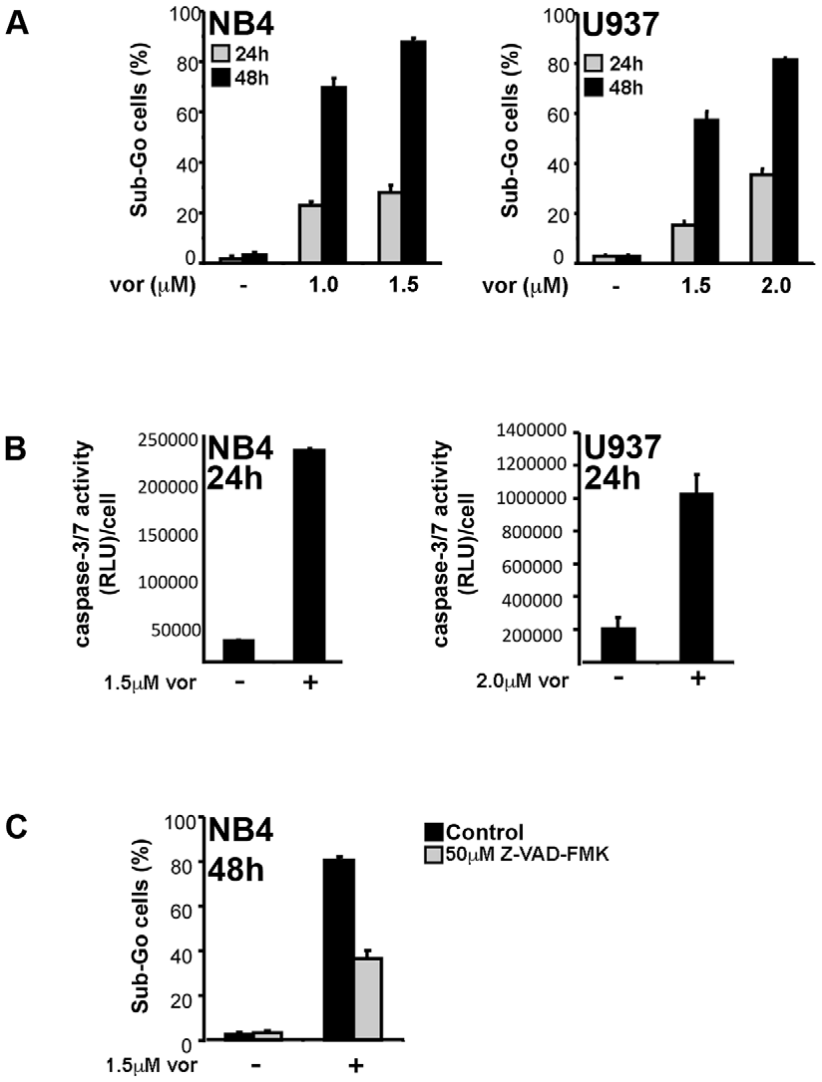
Vorinostat induces apoptosis in AML cells following the induction of DNA damage and cell cycle arrest. NB4 and U937 cells were treated with vorinostat and assayed for apoptosis at the indicated time points. **A** Cells were stained with PI and analyzed by flow cytometry. Apoptosis was quantified as the percentage of cells with DNA content below that of the Go-G1 peak (Sub-Go). **B** Cells were counted and then assayed for caspase-3/7 activity using a luciferase-based assay, where reactive light units (RLU) are normalized to number of cells. **C** Cells were pre-treated with 50 µM Z-VAD-FMK, a pan-caspase inhibitor for 1 h and then treated with vorinostat for 48 h. Apoptosis was then quantified as percentage of cells with Sub-Go DNA content.

To assay for cell death, NB4 and U937 cells were treated with vorinostat and subsequently stained with PI in order to quantify fragmented DNA by flow cytometry. Vorinostat induced cell death in a dose- and time-dependent manner in both NB4 and U937 cells beginning at 24 h (Figure 3A). As opposed to the induction of cell cycle arrest, which was observed after 6 h–12 h of vorinostat treatment, no significant cell death until at least 18 h post-treatment (data not shown). We next assessed caspase-dependency of cell death using PI staining and a caspase-3/7 activity assay. Vorinostat induced caspase-3/7 activity in NB4 and U937 cells after 24 h of treatment, consistent with the appearance of cell death (Figure 3B). Inhibition of caspase activity by the pan-caspase inhibitor Z-VAD-FMK, suppressed vorinostat-induced cell death (Figure 3C). Thus, DNA damage and G2-M arrest precedes vorinostat-induced apoptosis and this apoptosis is largely caspase-driven.

### Vorinostat induces reactive oxygen species and antioxidants reduce vorinostat-induced DNA damage and apoptosis

**Figure 4:**
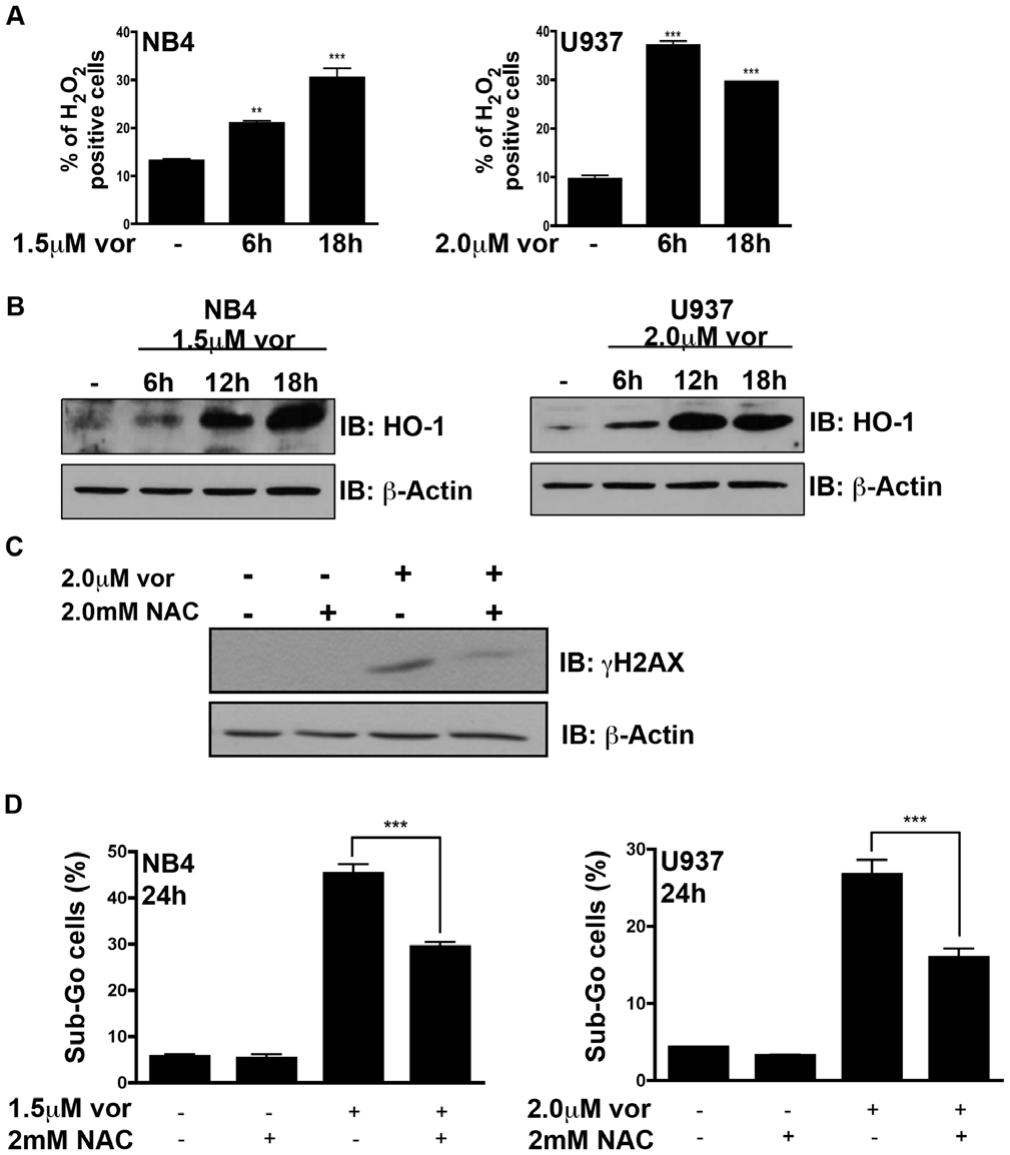
Vorinostat mediated DNA damage and apoptosis is in part due to reactive oxygen species. **A** NB4 and U937 cells were treated with vorinostat for 6 h and 18 h and stained with DCF-DA to quantify intracellular peroxides. **B** NB4 and U937 cells were treated with vorinostat for 6 h, 12 h and 18 h and induction of HO-1 was assessed by western blot. β-Actin was used as a loading control. **C** U937 cells were pre-treated with 2 mM N-acetyl cysteine (NAC), an antioxidant, followed by 2.0 µM vorinostat for 18 h. Induction of γH2AX was assessed by western blot. β-Actin was used as a loading control. **D** Cells were stained with PI after 24 h of exposure to vorinostat and/or N-acetyl cysteine, and apoptosis was quantified as percentage of cells with Sub-Go DNA content. Asterisks show significant difference (** <0.01 *** <0.001).

Previous studies have reported that HDACi treatment results in ROS production and ROS has subsequently been postulated to be a key mechanism in HDACi-induced apoptosis [3]. We analyzed the production of peroxides (mainly hydrogen peroxide) by DCF-DA staining in NB4 and U937 cells after treatment with vorinostat for 6 h or 18 h. Consistent with the induction of the oxidative stress DNA adduct 8-oxo-G (Figure 1D), we observe an increase in peroxides beginning at 6 h in both cell lines (Figure 4A). To validate this finding we assayed for HO-1 protein expression, a marker of cellular oxidative stress. Treatment of NB4 and U937 cells with vorinostat resulted in the up-regulation of HO-1 protein beginning at 6 h (Figure 4B). Augmented ROS levels can be due to a decrease in intracellular GSH, however, at a time point preceding the appearance of ROS, vorinostat was not found to alter GSH levels (Figure S1A).

If left unrepaired, DNA adducts including 8-oxo-G can give rise to DSBs, the most cytotoxic DNA lesion. To determine if ROS-induced DNA damage may contribute to vorinostat-induced apoptosis, NB4 cells were pre-treated with the antioxidant NAC for 1 h prior to treatment with vorinostat. Pre-treatment with this antioxidant resulted in a significant reduction in vorinostat-induced DSBs, as measured by γH2AX protein expression (Figure 4C). Pre-treatment with NAC was also associated with a reduction of cells arresting at the G2-M cell cycle phase (Figure S1B) and a partial protection in vorinostat-induced apoptosis (Figure 4D). In addition to NAC, the antioxidants L-GSH and MnTMPyP, a superoxide dismutase mimetic, also protected against vorinostat induced apoptosis in NB4 and U937 cells (Figure S1C).

### Vorinostat induces DNA damage in AML patient blasts and reduces their viability

**Figure 5:**
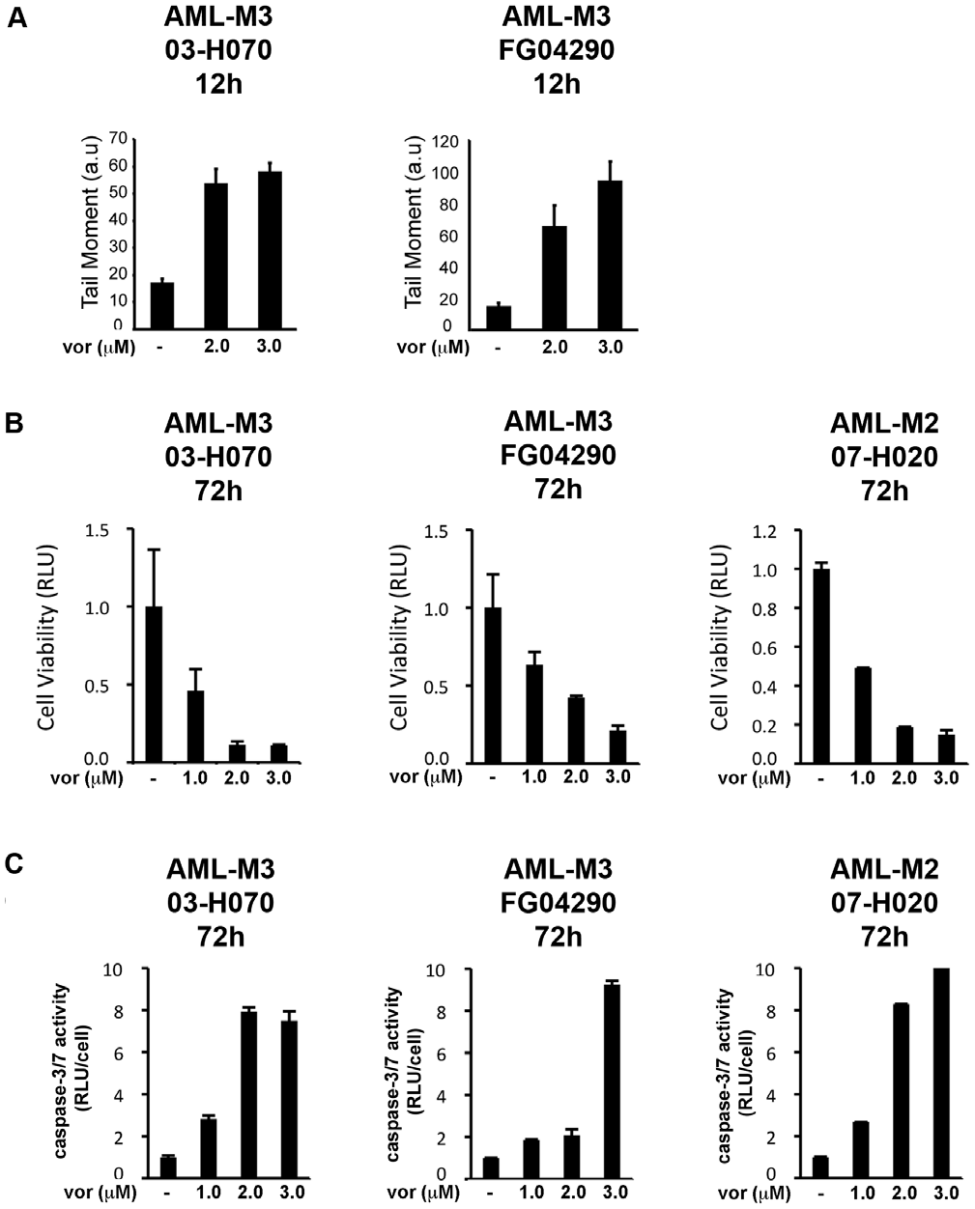
Vorinostat induces DNA damage and apoptosis at clinically achievable concentrations in AML-patient derived blasts cultured *ex vivo*. **A** Alkaline COMET assay measures DNA double strand breaks, single strand breaks and alkalile-labile sites. DNA damage is represented as an increase in Tail Moment (a.u.) after 12 h exposure to vorinostat. **B** Cells were assayed for viability 72 h post-vorinostat treatment using a luciferase based assay. **C** **C**aspase-3/7 activity (normalized to cell number) was assessed as a marker of apoptosis.

To extend our findings, leukemic blasts from AML patients were cultured and treated with vorinostat *ex vivo*, and DNA damage was assessed using the COMET assay. Vorinostat caused a significant increase in tail moment, beginning 12 h post vorinostat treatment (Figure 5A). The increase in DNA damage correlated with a decrease in cell viability (Figure 5B) and an increase in caspase-3/7 activity (Figure 5C), measured at a later time point (72 h). Blasts from an additional AML patient were assayed for caspase-3/7 activity in response to vorinostat and/or NAC. In these blasts, vorinostat caused a significant increase caspase-3/7 activity and pre-treatment with NAC abolished this increase (Figure S2). These data confirm that, as in our AML cell line models, treatment of AML patient blasts with vorinostat results in an early induction of DNA damage followed at later time points by a decrease in cell viability and apoptosis. In addition, they also suggest vorinostat-induced ROS as an important contributor to vorinostat-induced apoptosis and possibly DNA damage. Importantly, vorinostat did not induce DNA damage in PBMCs collected from healthy volunteers, as measured by alkaline comet assay (Figure S3A). Normal PBMCs also demonstrated a reduced sensitivity to vorinostat-induced loss of cell viability and apoptosis (Figure S3B, and C).

## Discussion

**Figure 6:**
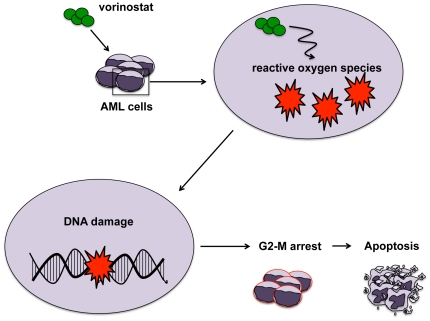
Treatment of acute myeloid leukemia cells with vorinostat results in an early induction of reactive oxygen species. The production of reactive oxygen species coincides with the appearance of DNA damage and partially contributes to the DNA damage accumulated. Reactive oxygen species production and accrual of DNA damage is followed by a G2-M cell cycle arrest and finally apoptosis at later time points.

HDACs are overexpressed in many tumor types and have been shown to play an important role in mediating aberrant epigenetic reprogramming, silencing tumor suppressor genes and favoring transformation [2], [27]. HDAC activity can be targeted using small molecule HDACi. Currently, two HDACi (vorinostat and romidepsin) have garnered FDA approval, both for the treatment of CTCL [28], [29]. One manner by which HDACi have been proposed to induce cell death is through the induction of DNA damage and/or genomic instability. In this work, we have performed a careful analysis of a clinically relevant HDAC inhibitor, vorinostat, on DNA damage, cell growth and apoptosis. For the first time, we show that vorinostat induces DNA damage followed by a G2-M arrest (Figure 6). This DNA damage correlates with increased apoptosis in leukemic cell lines (Figure 6) and, importantly, increased apoptosis in primary leukemic blasts.

Vorinostat is FDA approved for the treatment of CTCL, where it is administered at a dose of 400 mg once daily [29]. In CTCL patients this regimen resulted in a maximum concentration 1.2±0.53 µM. The same regimen in patients with advanced solid malignancies resulted in a maximum concentration of 1.81±0.70 µM [30]. Recently, maximum concentrations of 4.80±2.8 µM vorinostat have been achieved in patients using a pulse dose approach [31]. The doses used in this study to induce DNA damage and apoptosis in AML cells are within the clinically achievable range. At lower doses vorinostat does not induce apoptosis but rather growth arrest and differentiation [20]. We speculate that the DNA damage inflicted at the higher vorinostat doses is a sufficiently traumatic event to trigger apoptosis, and as such is an important factor determining whether the cells will growth arrest or die. Consistent with a previous report [16], we find DNA double strand breaks (DSBs) to be induced by vorinostat as early as 6 h (by neutral COMET assay) coinciding with the induction of γH2AX (Figure 1). The induction and persistence of DSBs we observe may be due to a number of mechanisms. For example, HDACi have previously been shown to induce DSBs or inhibit their repair by down-regulating expression of DNA repair proteins including NBS1, BRAC1/2, Ku70, Ku80 and RAD51 [15], [32], [33]. However, we were unable to detect any reduction in Ku70, RAD51 or NBS1 protein levels in vorinostat treated NB4 or U937 cells (Figure S4). This leads us to hypothesize that the DSBs may be due altered repair protein function [14] or modulation of the DNA damage “histone-code” [34], [35], [36]. It is worth noting that malignant cells have been shown to be more sensitive to HDACi induced DNA damage and apoptosis than non-malignant cells [37], [38], an observation that we confirmed in vorinostat-treated leukemic blasts, compared to normal PBMCs (Figure S3).

Of interest is a recent study where vorinostat treatment in MCF-7 breast cancer cells induced DSBs that were found to co-localize with replication factories and correlated with a slight decrease in DNA replication and a significant reduction in replication fork velocity [24]. Using similar methods (BrdU incorporation) we show the opposite observation that vorinostat increases DNA replication in NB4 and U937 cells. It is tempting to speculate that in our cell lines, the increase in DNA replication results in a greater amount of DNA damage compared to in MCF-7 cells, where vorinostat-induced damage results in the arrest of replication forks. In addition, MCF-7 cells arrest both in G1 and G2-M in response to vorinostat, possibly limiting the effect of vorinostat-induced DNA damage and cell death. Indeed, in these cells, higher doses of vorinostat (4–10 µM) are generally required to slow DNA replication, induce cell cycle arrest or apoptosis and even then the apoptosis observed is relatively modest [24], [39]. Interestingly, inhibiting the G1-phase arrest has been demonstrated to sensitize MCF-7 cell to HDAC inhibitor-induced apoptosis [40]. In contrast, NB4 and U937 cells are unable to mount a G1-phase arrest in response to vorinostat, possibly leading to a greater accumulation of DNA damage and more cell death. This is supported by the significant increase in γH2AX positive cells arresting in G2-M in response to vorinostat (Figure 2). It is also of general interest to mention that while capable of a G1-arrest [41], U937 cells do not arrest at the G1-phase in response to vorinostat, despite inducing expression of p21 (data not shown), a powerful inducer of the G1-phase arrest. Consistent with this observation, p21 expression does not coincide with a degradation of cyclin-E (Figure 2B).

A number of studies have reported that vorinostat induces ROS in leukemic cells [18], [42] and other malignant cell types [43], [44]. To expand on these findings, we found that vorinostat induced 8-oxo-G, a marker of oxidative DNA damage (Figure 1D). It should be noted that the increase in vorinostat-induced 8-oxo-G was statistically significant in U937 but not NB4 cells. 8-oxo-G represents a single oxidative lesion and vorinostat may induce other oxidative lesions like 2,6-diamino-4-hydroxy-5-formamidopyrimidine, 3-methyladenine and/or 7-methyladenine to a greater extent in NB4 cells. The ability of vorinostat to induce such oxidative lesions will be assessed in future studies. The persistence of such damaged bases can lead to increasingly cytotoxic lesions such as DSBs [45]. Indeed, pre-treatment with NAC greatly diminished vorinostat-induced γH2AX, resulting in both a reduced G2-M phase arrest and apoptosis (Figure 4). However, the reduction in apoptosis by NAC is modest and highlights the possibly pleiotrophic mechanisms of vorinostat-induced DNA damage and apoptosis. Indeed, HDACi have been demonstrated to activate both death receptor (extrinsic) and mitochondrial (intrinsic) apoptotic pathways. HDACi have been shown to up-regulate death ligands TRAIL, TRAIL-R2, Fas, Fas-L and TNFα of the extrinsic pathway [3]. HDACi have also been shown to induce pro-apoptotic genes (i.e. Bmf, Bim, Bax, Puma, Noxa) and down-regulate pro-survival genes (i.e. survivin, XIAP) favoring initiation of the intrinsic apoptotic program [3].

Finally, we investigated DNA damage in primary leukemic blasts. Consistent with our findings in cell lines, vorinostat-induced DNA damage could be measured at an early time point (Figure 5A). The induction of DNA damage by vorinostat was also consistent with a reduction in cell viability and an increase in caspase-3/7 activity (Figure 5B, 5C). It is interesting to note that while patient-derived leukemia blasts cultured *ex vivo* do not cycle, DNA damage is still induced in response to vorinostat. It is possible that in the context of this model, vorinostat-induced DNA damage is replication-independent and transcription-dependent, as previously demonstrated by Conti *et al.* [24].

In the clinic, the results of vorinostat monotherapy have been mixed [46], [47]. Our data demonstrate that DNA damage is a relevant mechanism of vorinostat-induced cytotoxicity in AML cells. We propose the assessment of DNA damage in patients treated with vorinostat to ascertain whether the induction of DNA damage correlates with response. Our findings suggest that AML patients unable to mount a G1-phase arrest to vorinostat may respond favorably. Indeed, future directions will be aimed at understanding the mechanisms by which vorinostat bypass the G1-phase arrest in our cell line models. The existence of numerous mechanisms by which vorinostat promotes genomic instability suggests the combination of vorinostat with other DNA damaging agents, or possibly G1-arrest inhibitors. Indeed, vorinostat along with other HDACi have been demonstrated to enhance sensitivity to ionizing radiation and DNA damaging drugs such as mitomycin C, cisplatin, bleomycin, doxorubicin, etoposide, 5-fluorouracil, Ara-C and topotecan [48], [49]. Alternatively, the many mechanisms of vorinostat may complicate more precisely targeting a desired cellular function, thereby lessening its therapeutic efficacy. For example, the vorinostat target HDAC3 has been meticulously demonstrated in knockout and knockdown experiments to be vital to DSB repair [24], [50]. Thus, the development of more specific HDACi that target a single HDAC class or isoform may be a promising approach to improve efficacy of this novel class of anti-cancer agents.

## Supporting Information

Figure S1 **Vorinostat does not alter intracellular GSH, and antioxidants reduce vorinostat-induced G2-M arrest and apoptosis.** **A** GSH levels were quantified after a 4 h treatment with vorinostat in NB4 and U937 cells. BSO inhibits GSH synthesis and was used as a control. **B** NB4 and U937 cells were pre-treated for 1 h with NAC followed by vorinostat treatment for 18 h. Afterwards, the cells were stained with propidium iodide to measure DNA content. **C** NB4 and U937 cells were pre-treated for 1 h with the antioxidant L-GSH or the SOD mimetric MnTMPyP for 1 h followed by vorinostat treatment for 24 h. Cells were then stained with propidium iodide to quantify DNA fragmentation. Asterisks indicate a significant difference (*** <0.001).
(TIF)

Figure S2 **The antioxidant NAC protects against vorinostat-induced caspase-3/7 activity.** Blasts from an AML patient were treated with vorinostat and/or NAC for 72 h at which point caspase-3/7 activity was quantified. Asterisks indicate a significant difference and a non-significant difference is indicated as “n.s”. (*** <0.001).
(TIF)

Figure S3 **Normal PBMCs are less sensitive to vorinostat-induced DNA damage and apoptosis.** PBMCs were collected from healthy volunteers and treated with vorinostat. **A** Alkaline COMET assay to measure DNA double strand breaks, single-strand breaks and alkalile-labile sites. At least 100 nuclei were randomly selected and quantified for DNA damage, represented as an increase in Tail Moment (a.u). **B** Cells were stained with PI and analyzed by flow cytometry. Apoptosis was quantified as the percentage of cells with DNA content below that of the Go-G1 peak (Sub-Go). **C** Cells were treated with vorinostat for 72 h and assayed for viability using a luciferase based assay. **D** Caspase-3/7 activity (normalized to cell number) was assessed after 72 h as a marker of apoptosis. Asterisks indicate a significant difference and a non-significant difference is indicated as “n.s”. (*** <0.001).
(TIF)

Figure S4 **Vorinostat has no effect on DNA repair protein expression.** Protein levels for the DNA repair proteins Ku70, RAD51 and NBS1 were assayed by western blot. β-Actin was used as a loading control.
(TIF)
