# Supplementary figures and images for: Vorinostat Induces Reactive Oxygen Species and DNA Damage in Acute Myeloid Leukemia Cells
Source: PLoS One. 2011 Jun 10;6(6):e20987. doi: 10.1371/journal.pone.0020987 (PMC3112218; doi:10.1371/journal.pone.0020987)

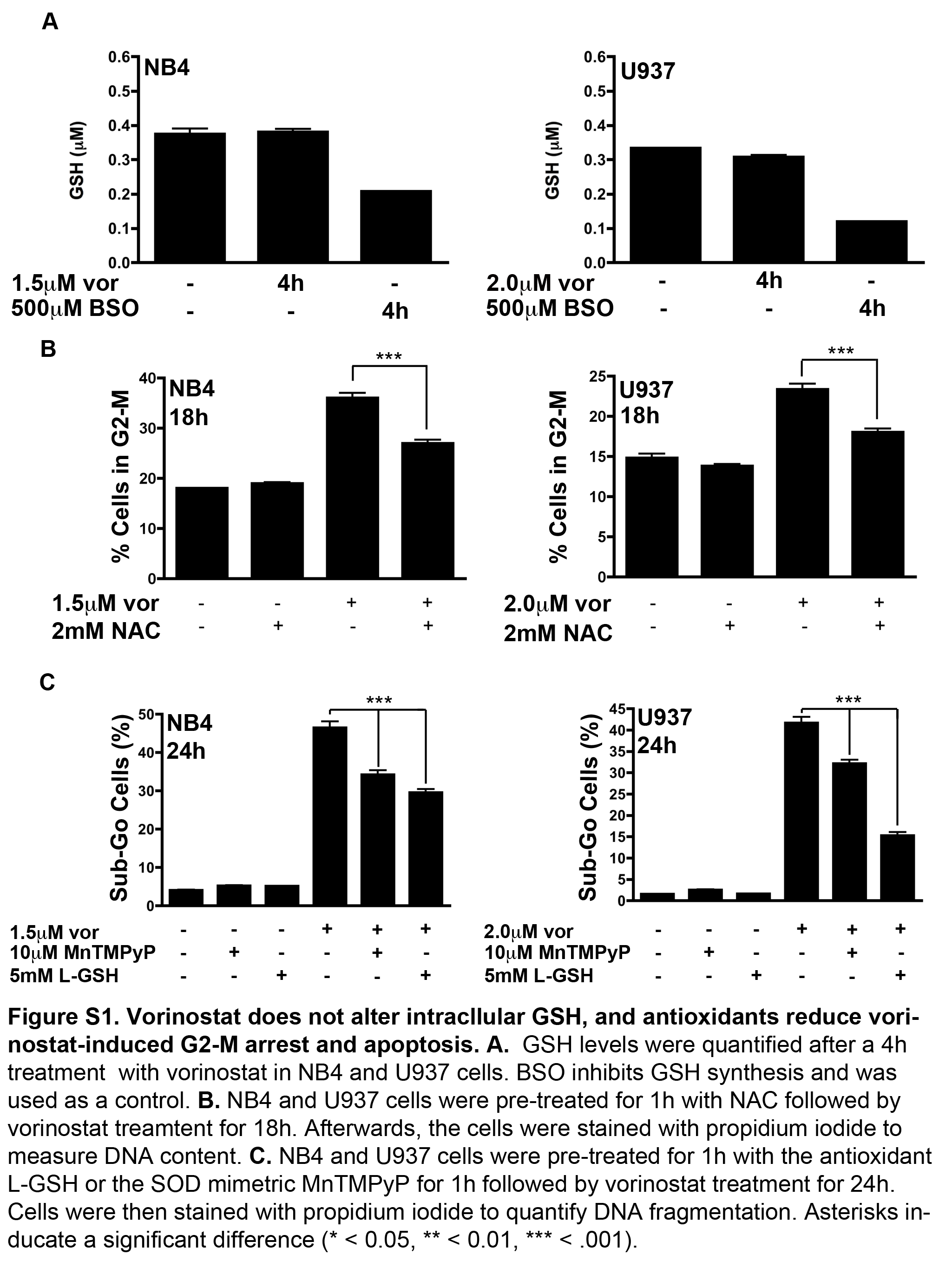

Supplement: Figure S1 — Vorinostat does not alter intracellular GSH, and antioxidants reduce vorinostat-induced G2-M arrest and apoptosis. A GSH levels were quantified after a 4 h treatment with vorinostat in NB4 and U937 cells. BSO inhibits GSH synthesis and was used as a control. B NB4 and U937 cells were pre-treated for 1 h with NAC followed by vorinostat treatment for 18 h. Afterwards, the cells were stained with propidium iodide to measure DNA content. C NB4 and U937 cells were pre-treated for 1 h with the antioxidant L-GSH or the SOD mimetric MnTMPyP for 1 h followed by vorinostat treatment for 24 h. Cells were then stained with propidium iodide to quantify DNA fragmentation. Asterisks indicate a significant difference (*** <0.001). (TIF) [file pone.0020987.s001.tif]

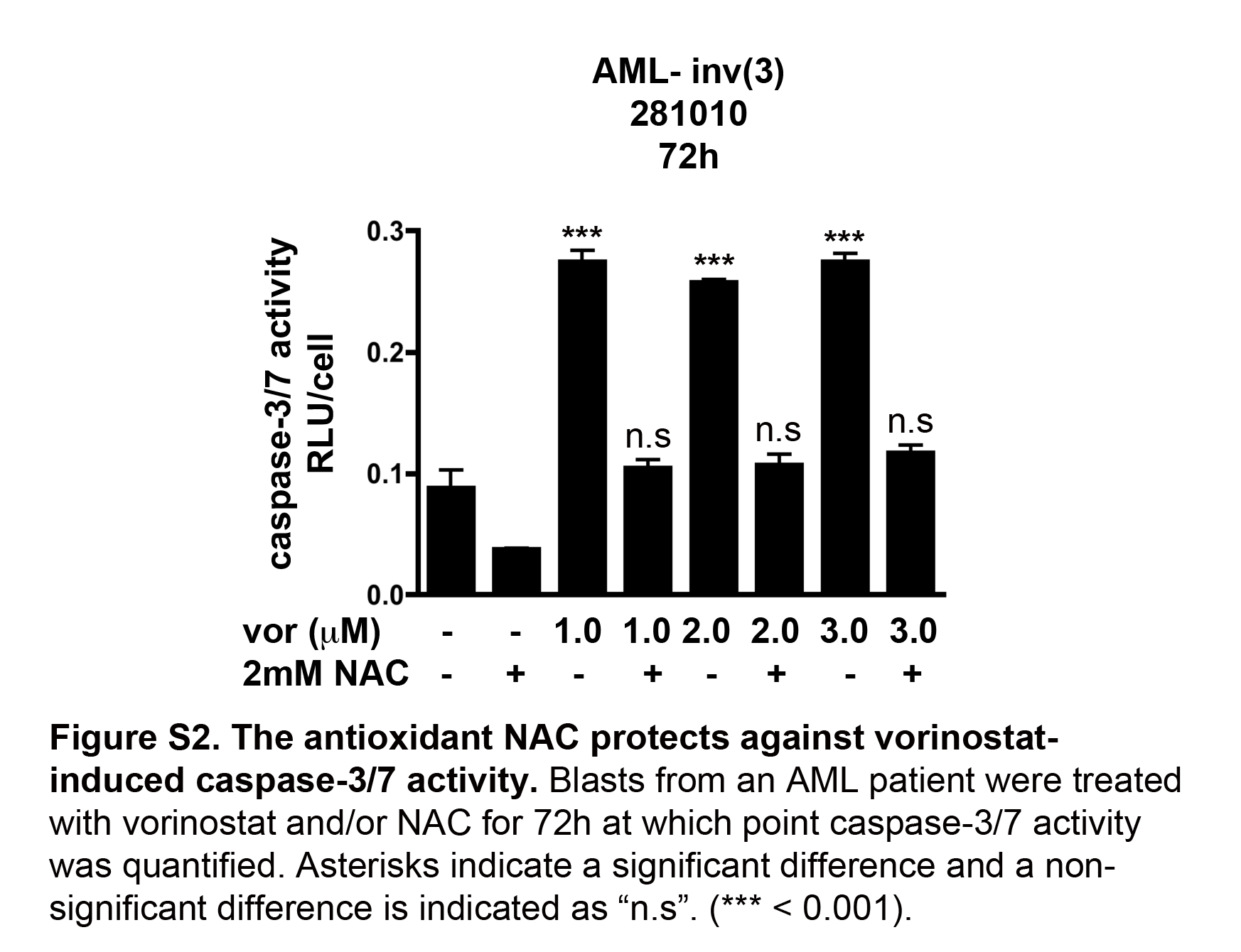

Supplement: Figure S2 — The antioxidant NAC protects against vorinostat-induced caspase-3/7 activity. Blasts from an AML patient were treated with vorinostat and/or NAC for 72 h at which point caspase-3/7 activity was quantified. Asterisks indicate a significant difference and a non-significant difference is indicated as “n.s”. (*** <0.001). (TIF) [file pone.0020987.s002.tif]

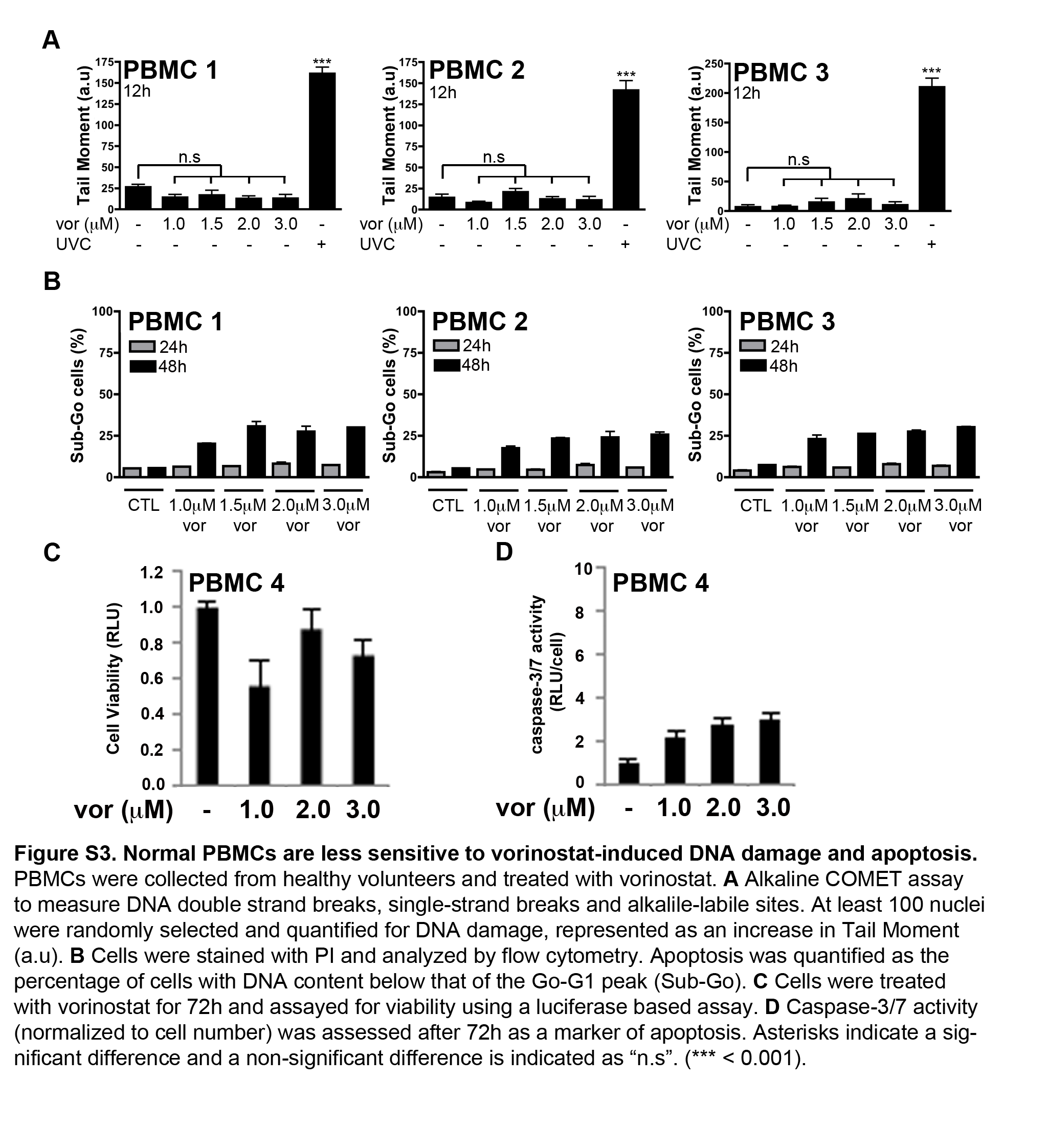

Supplement: Figure S3 — Normal PBMCs are less sensitive to vorinostat-induced DNA damage and apoptosis. PBMCs were collected from healthy volunteers and treated with vorinostat. A Alkaline COMET assay to measure DNA double strand breaks, single-strand breaks and alkalile-labile sites. At least 100 nuclei were randomly selected and quantified for DNA damage, represented as an increase in Tail Moment (a.u). B Cells were stained with PI and analyzed by flow cytometry. Apoptosis was quantified as the percentage of cells with DNA content below that of the Go-G1 peak (Sub-Go). C Cells were treated with vorinostat for 72 h and assayed for viability using a luciferase based assay. D Caspase-3/7 activity (normalized to cell number) was assessed after 72 h as a marker of apoptosis. Asterisks indicate a significant difference and a non-significant difference is indicated as “n.s”. (*** <0.001). (TIF) [file pone.0020987.s003.tif]

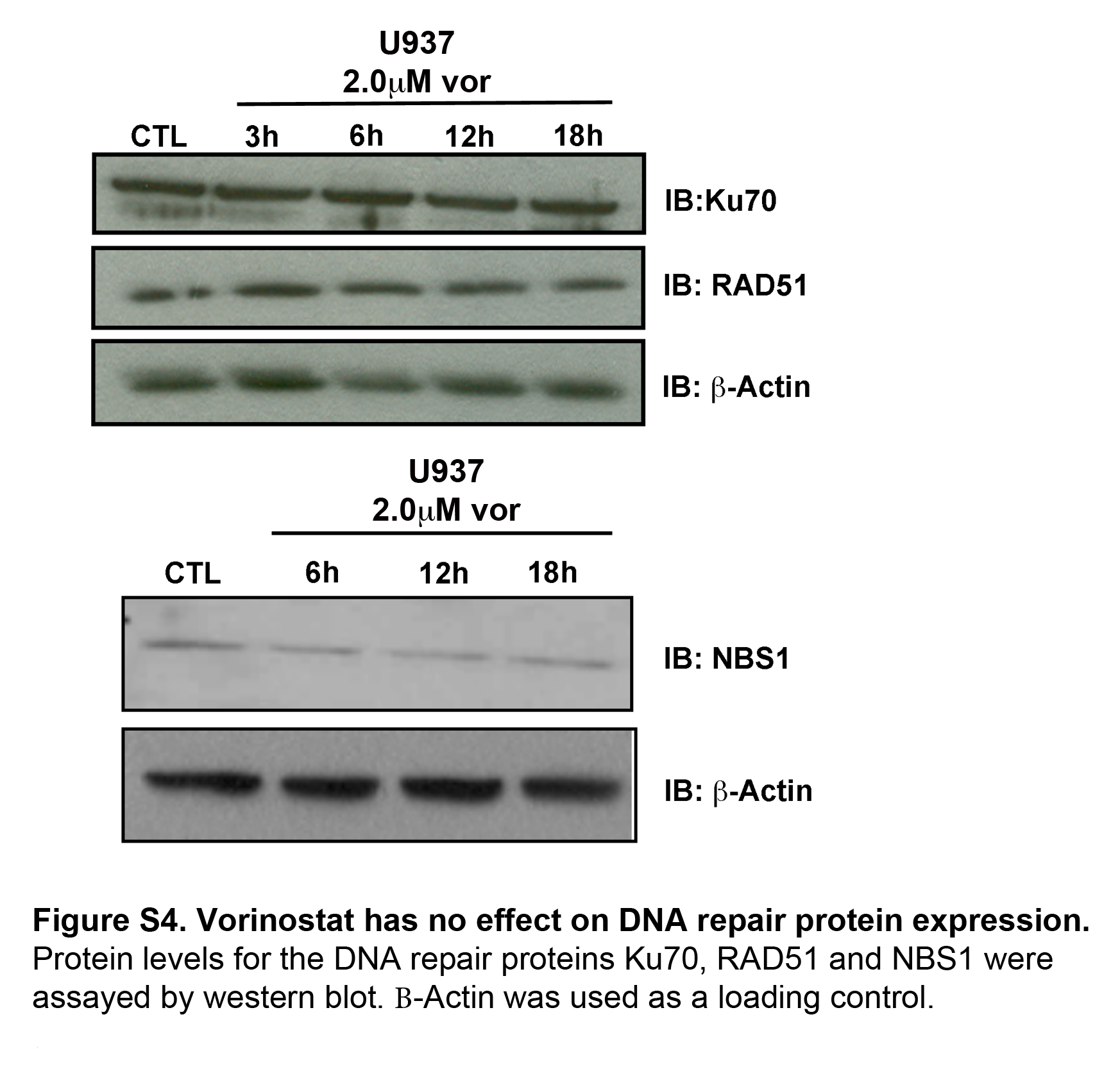

Supplement: Figure S4 — Vorinostat has no effect on DNA repair protein expression. Protein levels for the DNA repair proteins Ku70, RAD51 and NBS1 were assayed by western blot. β-Actin was used as a loading control. (TIF) [file pone.0020987.s004.tif]
